# Isolation and cDNA cloning of four peptide toxins from the sea anemone *Heteractis aurora*

**DOI:** 10.1590/1678-9199-JVATITD-2024-0019

**Published:** 2024-10-28

**Authors:** Tomohiro Homma, Masami Ishida, Yuji Nagashima, Kazuo Shiomi

**Affiliations:** 1 Department of Marine Biology and Sciences, School of Biological Sciences, Tokai University, Sapporo, Japan.; 2 Department of Ocean Sciences, Tokyo University of Marine Science and Technology, Tokyo, Japan.; 3 Department of Agro-Food Science, Niigata Agro-Food University, Niigata, Japan.; 4 Department of Food Science and Technology, Tokyo University of Marine Science and Technology, Tokyo, Japan.

**Keywords:** Crab toxicity, Heteractis aurora, Peptide toxin, Sea anemone

## Abstract

**Background:**

Sea anemones are well known to contain multiple peptide toxins. However, of more than 1100 species of sea anemones distributed worldwide, only a little over 50 have been studied for peptide toxins. Therefore, innumerable unique and novel peptide toxins remain to be discovered in unstudied sea anemones.

**Methods:**

Isolation of peptide toxins in the sea anemone *Heteractis aurora* was attempted by gel filtration and reverse-phase high performance liquid chromatography, using the toxicity to crabs as an index. The amino acid sequences of the isolated four toxins (Hau I-IV) and their precursors were determined using a combination of protein sequencing and cDNA cloning.

**Results:**

Hau I and IV were potently lethal to crabs, whereas Hau II and III were only paralytic. The precursor proteins of the four toxins were commonly composed of a signal peptide, a propart, and the remaining region including a mature peptide. Interestingly, four and two copies of the mature peptide were present in the precursor proteins of Hau II and III, respectively. Homology searches revealed that Hau I (30 amino acid residues) is a novel peptide toxin, although it has the same cysteine pattern CXXC-C-C as the boundless β-hairpin (BBH) family. Hau II (27 amino acid residues) and III (28 amino acid residues) were homologous with the BBH family, whereas Hau IV (49 amino acid residues) was a new member of the well-known type 1 sodium channel toxin family.

**Conclusion:**

This study showed that a novel class of toxin (Hau I), two BBH family toxins (Hau II and III), and a type 1 sodium channel toxin (Hau IV) are present in the toxin of the sea anemone *H. aurora*.

## Background

Sea anemones are venomous cnidarians belonging to the order Actiniaria. In common with other venomous cnidarians such as jellyfish and stinging corals, they possess a complex cocktail of toxins in specialized stinging organelles (called nematocysts) distributed throughout their bodies to capture preys and/or protect themselves from predators [1, 2]. However, unlike other venomous cnidarians whose toxins are primarily proteinaceous, the main toxic components in sea anemones are peptides that are diverse in structure and toxicity [1-5]. In fact, 697 toxins from 55 species of sea anemones are included in the National Center for Biotechnology Information (NCBI) protein database, of which 420 (approximately 60%) are peptides (as of July 31, 2024). 

Most of known sea anemone peptide toxins are divided into two classes of neurotoxins, sodium channel toxins [6-12] and potassium channel toxins [7, 11 13]. Based on the determined amino acid sequences, the sodium channel toxins can be grouped into three types: type 1 and 2 toxins are commonly composed of 46-51 amino acid residues cross-linked by three disulfide bridges but are distinguished from each other in the entire sequence; and type 3 toxins are shorter peptides with 27-32 amino acid residues and three or four disulfide bridges. Calitoxins I and II, both of which have 46 amino acid residues and three disulfide bridges, are also known as sodium channel toxins distinct from types 1-3 toxins [14, 15] and classified as type 4 sodium channel toxins [12]. On the other hand, the potassium channel toxins were initially divided into three types: type 1 toxins have 35-37 amino acid residues and three disulfide bridges; type 2 toxins are similar to Kunitz-type protease inhibitors, having 58 or 59 amino acid residues and three disulfide bridges; and type 3 toxins have 41 or 42 amino acid residues and three disulfide bridges. Subsequently, structurally novel potassium channel toxins have been isolated from sea anemones and added as type 4-6 toxins to the potassium channel toxin family as follows: type 4 toxins, SHTX I and II (28 amino acid residues and two disulfide bridges) from *Stichodactyla haddoni* [16]; type 5 toxin, BcsTx3 (50 amino acid residues and four disulfide bridges) from *Bunodosoma caissarum* [17]; and type 6 toxin, Ate1a (only 17 amino acid residues and two disulfide bridges) from *Actinia tenebrosa* [18]. 

In addition to the two classes of the peptide toxins described above, structurally and/or functionally unique peptide toxins have also been isolated from some sea anemone species [19]. For example, gigantoxin I, isolated from *Stichodactyla gigantea*, is the first epidermal growth factor-like toxin [20] that can indirectly inactivate the transient receptor potential vanilloid subtype I channels [21]. Other examples are APETx2 from *Anthopleura elegantissima* [22] and PhcrTx1 from *Phymanthus crucifer* [23]. These two peptide toxins are structurally dissimilar but act similarly on acid-sensing ion channels. Importantly, recent transcriptomic and/or proteomic studies on some sea anemones, such as *Exaiptasia diaphana* [24], *Heteractis crispa* [25], *Heteractis magnifica* [26], and *Entacmaea quadricolor* [27], have revealed that sea anemones, regardless of species, contain a variety of peptides, most of which are likely to function as toxins [5]. However, only a little over 50 species of sea anemones have so far been studied for peptide toxins, although more than 1100 valid species are distributed worldwide [1]. Given these circumstances, it is very likely that a vast number of unique peptide toxins, which could serve as pharmacological reagents and/or lead compounds for therapeutic drugs, lie dormant in unstudied sea anemones.

The sea anemone *Heteractis aurora*, a member of the family Stichodactylidae, is a relatively large species (up to 25 cm in diameter of disc) inhabiting tropical or subtropical waters. Some biological activities, such as analgesic and central nervous system depressant activities, have been detected in the venom of *H. aurora* [28]; however, the substances responsible for these activities have not been identified. In our preliminary experiments, the crude extract of *H. aurora* was found to exhibit crab toxicity, suggesting the presence of peptide toxins. This prompted us to isolate peptide toxins from *H. aurora* and elucidate their amino acid sequences. We report here the isolation and molecular cloning of the following four peptide toxins from *H. aurora*: one novel class of toxin with the same cysteine pattern (CXXC-C-C) as the boundless β-hairpin (BBH) family [2] (corresponding to toxins called structural 9a toxins [4]), two BBH family toxins, and one type 1 sodium channel toxin. According to the nomenclature proposed by Oliveira *et al.* [3], the four toxins could be named U-SHTX-Hau1a, U-SHTX-Hau2a, U-SHTX-Hau2b, and δ-SHTX-Hau3a. However, three of the four toxins must be assigned the activity descriptor “U” to indicate unknown function. To avoid the use of “U” as much as possible, the four toxins are tentatively called Hau I-IV until their molecular targets are elucidated.

## Methods

### Sea anemone

Two *H. aurora* specimens (10 and 180 g in body weight) collected along the coast of Miyako Island, Okinawa Prefecture, were purchased from a retail aquarium shop in Osaka City. The specimens were transported alive to the laboratory. The smaller specimen was stored at -20° C until use for extraction, whereas the larger specimen was immediately cut into small pieces, frozen in liquid nitrogen, and stored at -80° C until use for cDNA cloning.

### Isolation method

The frozen specimen was thawed and, without being divided into parts, macerated in a motor using a pestle. A 5 g-portion of the macerate was homogenized in 25 mL of distilled water and centrifuged at 18800 × g and 4°C for 15 min. The supernatant (crude extract) was applied to gel filtration on a Sephadex G-50 column (2.5 × 90 cm; GE Healthcare, Piscataway, NJ, USA). Elution of the column was performed at a flow rate of approximately 40 mL/h using 0.01 M phosphate buffer (pH 7.0) containing 0.15 M NaCl. Fractions of 8 mL were collected and measured for absorbance at 280 nm and crab toxicity. Toxic fractions were combined, passed through a 0.45-µm pore size filter (DISMIC-25cs; Advantec, Tokyo, Japan), and then subjected to reverse-phase high performance liquid chromatography (HPLC) on a TSKgel ODS-120T column (0.46 × 25 cm; Tosoh, Tokyo, Japan). The column was first washed with 0.1% trifluoroacetic acid (TFA) and then eluted in two steps with a linear gradient of acetonitrile (0-14% in 5 min and 14-42% in 60 min) in 0.1% TFA at a flow rate of 1 mL/min. Peptides were monitored at 220 nm using a UV detector. The eluate corresponding to each peak was collected manually and assayed for crab toxicity. Thus, four peptide toxins, Hau I-IV (named in the order of elution), were isolated. 

### Crab toxicity assay

Crab toxicity was assayed using freshwater crabs (*Geothelphusa dehaani*), weighing approximately 5 g, purchased from the Tokyo Central Wholesale Market. Sample solutions were injected into the body cavity of crabs at 10 µL/g of crab body weight through the junction between the body and the leg. Symptoms induced in crabs were carefully observed for up to 2 h after injection. To determine the specific toxicity (lethal activity [LD50] and paralytic activity [ED50]) against crabs, groups of five crabs were administered with various doses of the toxin. Based on the rates of death and paralysis induced within 2 h, LD_50_ and ED_50_ were respectively calculated using the method described by Litchfield and Wilcoxon [29].

### Chemical analysis

Peptides were determined by the method of Lowry *et al.* [30] using bovine serum albumin as a standard. Molecular mass determination was performed by matrix assisted laser desorption ionization/time-of-flight mass spectrometry (MALDI/TOFMS) using a Shimadzu/Kratos Kompact MALDI I instrument (Shimadzu, Kyoto, Japan). Sinapinic acid was used as the matrix. Amino acid sequencing was carried out using an automatic gas-phase protein sequencer (LF-3400D TriCart with high sensitivity chemistry; Beckman Coulter, Fullerton, CA, USA).

### cDNA cloning

As described below, the cDNAs coding for Hau I-III were cloned by a combination of 3’ rapid amplification of cDNA ends (3’RACE) and 5’RACE. In the case of Hau IV, 5’RACE was unsuccessful; therefore, its cDNA was isolated by a combination of 3’RACE and reverse transcription polymerase chain reaction (RT-PCR). The designations and nucleotide sequences of the primers used in this study are listed in Table 1. The degenerate primers used for 3’RACE were designed based on the N-terminal amino acid sequences determined by protein sequencing and the gene-specific primers used for 5’RACE based on the partial nucleotide sequences determined by 3’RACE. In RT-PCR for Hau IV, the gene-specific forward primer (IV-Gsp-F1) was designed based on the nucleotide sequence of the 5’-untranslated region of the cDNA coding for Am III (type 1 sodium channel toxin) from *Antheopsis maculata* [31] and the gene-specific reverse primer (IV-Gsp-R1) based on the partial nucleotide sequences determined by 3’RACE. Throughout this study, PCR was carried out using Ex Taq polymerase (Takara, Kyoto, Japan) under the following conditions: 94°C for 5 min; 35 cycles of 94°C for 30 sec, 55°C for 30 sec, and 72°C for 1 min; and 72°C for 5 min.

**Table 1.** Designations and nucleotide sequences of the primers designed in this study

Experiment	Designation of primera	Nucleotide sequence of primer	Corresponding nucleotide sequenceb
3’RACE	I-Deg-F1	5’-GYCCIMGITGYCAYMGIMGIGAYC-3’	204-227
	I-Deg-F2	5’-GAYCAYTTYGGIAARTGYMGIAARYT-3’	224-249
	II-Deg-F1	5’-AYGTIGCIGTICCICCITGYGGIG-3’	180-203
	II-Deg-F2	5’-GYTAYCARCARGTIGGIAAYACITG-3’	207-231
	III-Deg-F1	5’-AYATHATHAAYCCICCITGYATHGG-3’	180-204
	III-Deg-F2	5’-CICCITGYATHGGITGYTAYTAYCA-3’	192-216
	IV-Deg-F1	5’-TGYYTITGYGCIWSIGAYGGICC-3’	157-179
	IV-Deg-F2	5’-GAYGGICCIWSIGTICAYGGIAAYA-3’	172-196
5’RACE	I-Gsp-R1	5’-GGAAAGCTAGACTGTTTTTGG-3’	280-300
	I-Gsp-R2	5’-CCTATTATTTGTCAGGACAAGG-3’	254-275
	I-Gsp-R3	5’-GTTTCCGACACTTCCCAAAATG-3’	227-249
	II-Gsp-R1	5’-GGCTCGGGCTCTCTCTTTC-3’	261-279
	II-Gsp-R2	5’-GGGCTGGACAGAGACTTGG-3'	242-260
	II-Gsp-R3	5'-CACGGACACACGTATTGCC-3'	221-249
	III-Gsp-R1	5’-CCACATTTAAATTTATCATAGA-3’	339-360
	III-Gsp-R2	5’-GATGATGTTGGGCTTGGGC-3’	274-292
	III-Gsp-R3	5’-ACACATTCGTTGCCGACTTG-3’	215-234
RT-PCR	IV-Gsp-F1	5’-CATTCAACATCGTTCAAGCAG-3’	1-21
	IV-Gsp-R1	5’-CGATTTAAACCTCATGTTCAGT-3’	357-378

RACE: rapid amplification of cDNA ends; RT-PCR: reverse transcription PCR.

a I: primer for Hau I; II: primer for Hau II; III: primer for Hau III; IV: primer for Hau IV; Deg: degenerate primer; Gsp: gene-specific primer; F: forward primer; R: reverse primer. Refer to Figure 2 for nucleotide sequences.

b Refer to Figure 2 for the positions of the nucleotide sequences.

Total RNA was extracted from 1 g of frozen samples with TRIzol reagent (Invitrogen, Carlsbad, CA, USA). For 3’RACE, first-stranded cDNA was synthesized from 5 µg of total RNA using a 3’RACE System for Rapid Amplification of cDNA Ends Kit (Invitrogen) and the oligo dT-adapter primer. The first 3’RACE reaction was performed using a degenerate primer (I-Deg-F1, II-Deg-F1, III-Deg-F1, or IV-Deg-F1) and the abridged universal amplification primer (AUAP; 5’-GGCCACGCGTCGACTAGTAC-3’) and the second 3’RACE reaction using a degenerate primer (I-Deg-F2, II-Deg-F2, III-Deg-F2, or IV-Deg-F2) and AUAP. The secondary PCR products were subcloned into the pT7Blue T-vector (Novagen, Darmstadt, Germany) and nucleotide sequences were determined using a Cy5 Thermo Sequenase Dye Terminator Kit (GE Healthcare) and a Long-Read Tower DNA sequencer (GE Healthcare). Based on the determined partial nucleotide sequences, the remaining 5’-terminal sequences were analyzed by 5’RACE as follows. First-stranded cDNA was synthesized from 5 µg of total RNA using a 5’RACE System for Rapid Amplification of cDNA Ends Kit (Invitrogen) and a gene-specific primer (I-Gsp-R1, II-Gsp-R1, or III-Gsp-R1). The first 5’RACE reaction was completed using a gene-specific primer (I-Gsp-R2, II-Gsp-R2, or III-Gsp-R2) and the abridged anchor primer (5’-GGCCACGCGTCGACTAGTACGGGGGGGGGGGGGGGG-3’), followed by reamplification of the PCR products using a gene-specific primer (I-Gsp-R3, II-Gsp-R3, or III-Gsp-R3) and AUAP. The secondary PCR products were subcloned into the pT7Blue T-vector and sequenced. RT-PCR for Hau IV was performed using a combination of the gene-specific primers, IV-Gsp-F1 and IV-Gsp-R1, and amplified products were subcloned into the pT7Blue T-vector and sequenced. 

## Results

### Isolation of toxins

**Figure 1.**
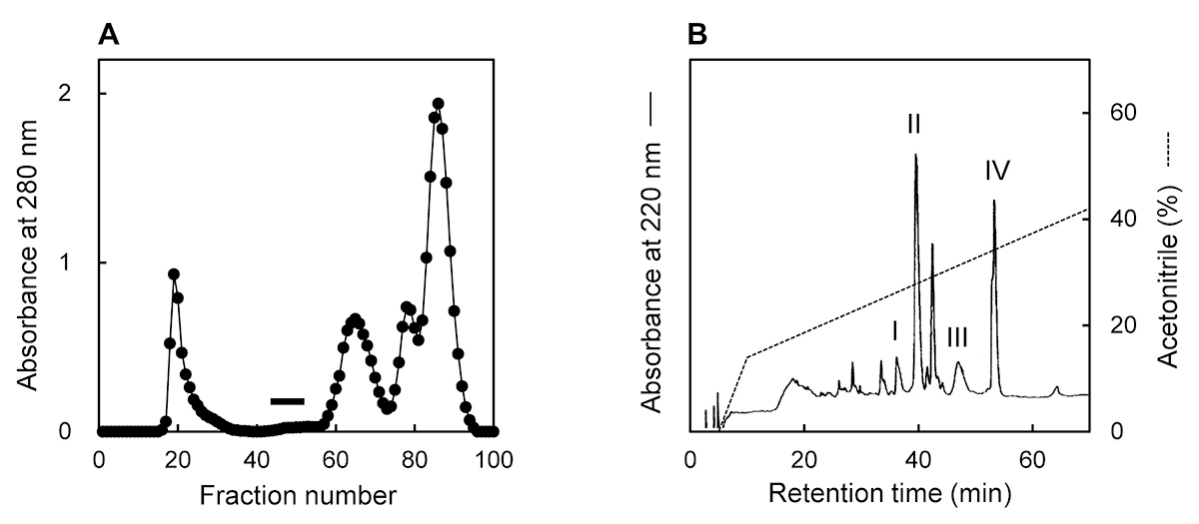
Isolation of Hau I-IV from *H. aurora*. **(A)** Gel filtration. Sample, crude extract; column, Sephadex G-50 (2.5 × 90 cm); solvent, 0.01 M phosphate buffer (pH 7.0) containing 0.15 M NaCl; volume per fraction, 8 mL. Toxic fractions - fractions 44 to 52 - are indicated by a bar. **(B)** Reverse-phase HPLC. Sample, toxic fractions obtained by gel filtration; column, TSKgel ODS-120T (0.46 × 25 cm); elution, linear gradient of acetonitrile in 0.1% TFA; flow rate, 1 mL/min. Hau I to Hau IV were eluted in labeled peaks.

Serial two-fold dilutions of the crude extract were prepared and tested for crab toxicity. Lethality was detected at up to a 32-fold dilution. In the gel filtration of the crude extract on a Sephadex G-50 column, four peaks with absorbance at 280 nm were observed and crab lethality was detected in fractions 44-52 between the first and second peaks (Figure 1 A ). The toxic fraction obtained by gel filtration was subjected to reverse-phase HPLC on a TSKgel ODS-120T column. Two lethal toxins, Hau I and IV, appeared at 36 and 53 min, respectively, and two paralytic toxins, Hau II and III, appeared at 39 and 47 min, respectively (Figure 1 B ). The starting material (5 g) yielded 54 µg of Hau I, 522 µg of Hau II, 305 µg of Hau III, and 495 µg of Hau IV. Hau I and IV exhibited lethal activity to crabs with LD_50_ of 14 and 53 µg/kg, respectively, whereas Hau II and III induced paralysis in crabs with ED_50_ of 2350 and 1400 µg/kg, respectively. 

Protein sequencing identified N-terminal 28, 24, 25, and 19 amino acid residues for Hau I, II, III, and IV, respectively. Notably, Hau I and II contained an unusual residue, hydroxy-Pro, at positions 5 and 6, respectively. In this study, the remaining amino acid sequences were elucidated using a cDNA cloning technique, not only because this technique provides valuable information about gene structures, but also because the cDNAs obtained could be used in the future to express toxins and their mutants.

### Molecular cloning of Hau I-IV

**Figure 2.**
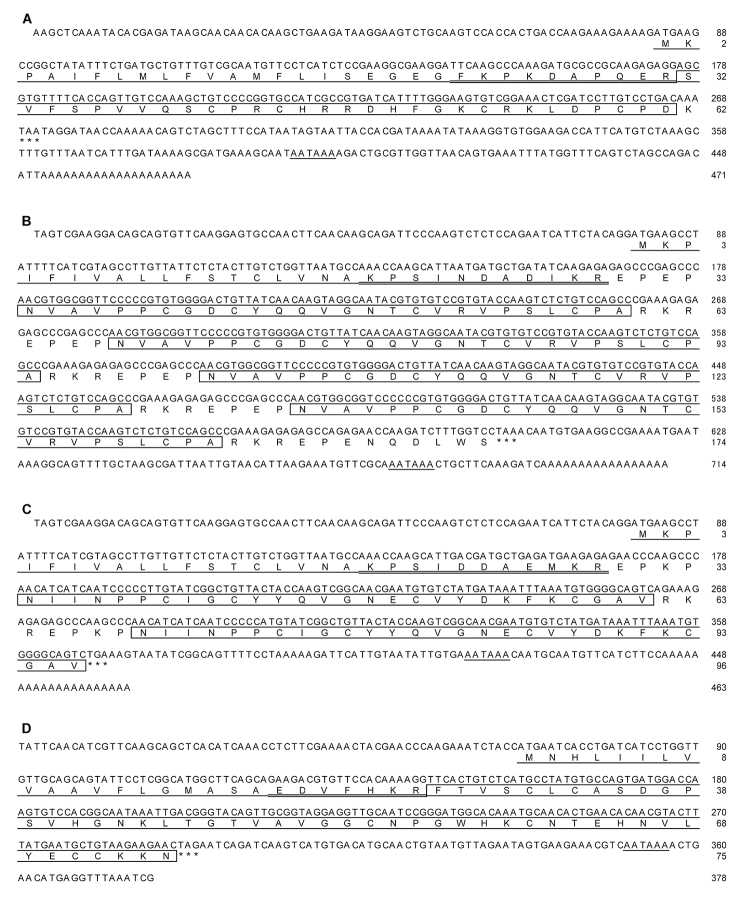
Nucleotide sequences of the cDNA coding for **(A)** Hau I, **(B)** Hau II, **(C)** Hau III, and **(D)** Hau IV. The deduced amino acid sequence is aligned below the nucleotide sequence. Nucleotide and amino acid numbers are shown at the right. Asterisks indicate a stop codon (TAA, TGA, or TAG). Putative signal sequences and poly (A) signals are singly underlined and putative proparts are doubly underlined. The sequences corresponding to the mature peptides are boxed. Accession numbers in the DDBJ/EMBL/GenBank databases: LC054037 for Hau I, LC054038 for Hau II, LC054039 for Hau III, and LC054040 for Hau IV.

As shown in Figure 2, the nucleotide sequences of the DNAs (471, 714, 463, and 378 bp for Hau I, II, III, and IV, respectively) coding for Hau I-IV were successfully determined by 3’RACE and 5’RACE (for Hau I-III) or 3’RACE and RT-PCR (for Hau IV). The established nucleotide sequences were deposited in the DDBJ/EMBL/GenBank databases under the following accession numbers: LC054037 for Hau I, LC054038 for Hau II, LC054039 for Hau III, and LC054040 for Hau IV. In common with the four cDNAs, a poly (A) signal (AATAAA) was recognized in the 3’-untranslated region. A poly (A) tail was also recognized in the 3’-untranslated region of the Hau I-III cDNAs. The open reading frames (coding for precursor proteins) seen in the Hau I-IV cDNAs were considerably different in length as follows: 186 bp (corresponding to 62 amino acid residues) in the Hau I cDNA, 522 bp (corresponding to 174 amino acid residues) in the Hau II cDNA, 288 bp (corresponding to 96 amino acid residues) in the Hau III cDNA, and 225 bp (corresponding to 75 amino acid residues) in the Hau IV cDNA. 

In the case of the precursor protein (62 amino acid residues) of Hau I, the region 1-21 was predicted to be a signal peptide using the SignalP program [32]. The region 32-59 matched the N-terminal sequence determined by protein sequencing, except for the residue at position 5 (Pro in cDNA cloning and hydroxy-Pro in protein sequencing). In addition, the molecular mass (3490.0) determined for Hau 1 by MALDI/TOFMS was close to that (3483.0) calculated for the region 32-61 after assuming the fifth residue to be hydroxy-Pro. Therefore, the region 32-61 was found to represent the mature Hau I peptide, implying that the C-terminal Lys in the precursor protein is removed through processing after translation. The remaining region 22-31 between the signal and mature peptides was assumed to be a propart, as recognized in a number of cDNAs encoding sea anemone peptide toxins.

The precursor protein (174 amino acid residues) of Hau II, similar to that of Hau I, was composed of a signal peptide (region 1-18), a propart (region 19-29), and the remaining segment (region 30-174). However, differing from the precursor protein of Hau I, four repeats of the same sequence (starting from Glu and ending with Arg) comprising 34 amino acid residues were observed in the region 30-165. The segment between Asn-5 and Ala-31 in the repeated sequence was concluded to correspond to the mature peptide of Hau II based on the following two facts: (1) the amino acid sequence ranging from Asn-5 to Leu-28 agreed with the N-terminal 24 residues determined for Hau II, except that the sixth residue from the N-terminus was Pro in cDNA cloning and hydroxy-Pro in protein sequencing, and (2) the molecular mass (2802.7) determined for Hau II by MALDI/TOFMS was close to that (2803.2) calculated for the segment 5-31 after assuming the sixth residue to be hydroxy-Pro. In conclusion, the region 30-174 in the precursor protein of Hau II contains four copies of the mature peptide (27 amino acid residues), each of which is preceded by the same four residues (Glu-Pro-Glu-Pro), followed by the same three residues (Arg-Lys-Arg) and nine C-terminal residues. 

The precursor protein (96 amino acid residues) of Hau III was similar to that of Hau II, being composed of a signal peptide (region 1-18), a propart (region 19-29), and the remaining portion (region 30-96) including two repeats of the same sequence (starting from Asn and ending with Val) comprising 28 amino acid residues. The first 25 amino acid residues of the repeated sequence were consistent with the N-terminal sequence determined for Hau III, and the molecular mass (3106.9) determined for Hau III by MALDI/TOFMS was close to that (3107.6) calculated for the repeated sequence. Therefore, the repeated sequence possibly represents a mature peptide. Of the two copies of the mature peptide, the N-terminal one (region 34-61) was preceded by four residues (Glu-Pro-Lys-Pro), followed by three residues (Arg-Lys-Arg), similar to Hau II, whereas the C-terminal one (region 69-96) was preceded by the same four residues but was not followed by any residues. 

The precursor protein (75 amino acid residues) of Hau IV was composed of a signal peptide (region 1-19), a propart (region 20-26), and the remaining segment (region 27-75) corresponding to the mature peptide. The region 27-75 had the same N-terminal sequence (up to 19 residues) as that determined for Hau IV, and the calculated molecular mass (5174.9) was close to the value (5177.4) determined for Hau IV by MALDI/TOFMS.

### Amino acid sequences of Hau I-IV

**Figure 3.**
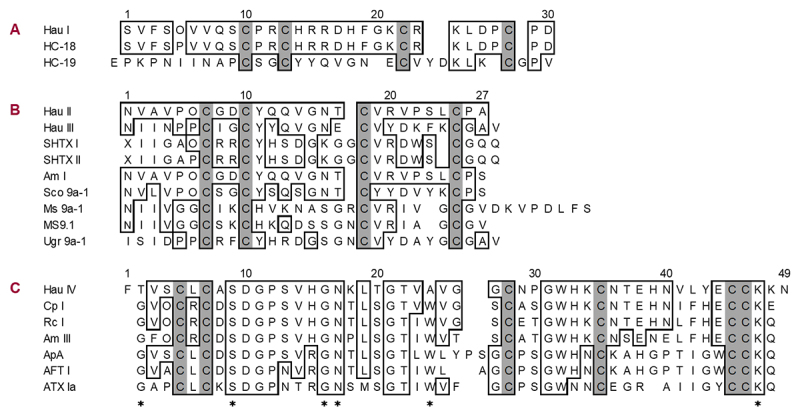
Amino acid sequence alignment of Hau I to Hau IV with analogous sea anemone peptide toxins. **(A)** Alignment of Hau I with two peptide toxins (HC-18 and HC-19) detected in *Heteractis crispa* [25]. Cysteine residues are shaded, a hydroxy-Pro residue is denoted by “O”, and identical residues with Hau I are boxed. Note that the only difference between Hau I and HC-18 at the fifth residue (hydroxy-Pro in Hau I and Pro in HC-18) is due to a post-translational modification; the two are completely identical. **(B)** Alignment of Hau II and III with members of the BBH family. SHTX I and II (type 4 potassium channel toxins) from *Stichodactyla haddoni* [16]; Am I (possibly sodium channel toxin) from *Antheopsis maculata* [31]; Sco 9a-1 (AnmTX Sco 9a-1; anti-inflammatory peptide) from *Stomphia coccinea* [33]; Ms 9a-1 (τ-AnmTX MS 9a-1; modulator of transient receptor potential ankyrin-repeat 1 [TRPA1]) and MS9.1 (AnmTX Ms 9a-2; possibly modulator of TRPA1) from *Metridium senile* [34]; Ugr 9a-1 (π-AnmTX Ugr 9a-1; acid-sensing ion channel 3 [ASIC3] inhibitor) from *Urticina grebelnyi* [35]. Cysteine residues are shaded, hydroxy-Pro residues are denoted by “O”, and identical residues with Hau II are boxed. **(C)** Alignment of Hau IV with type 1 sodium channel toxins. Cp I from *Condylactis passiflora* [36]; Rc I from *Radianthus crispus* [37]; Am III from *Antheopsis maculata* [31]; ApA from *Anthopleura xanthogrammica* [38]; AFT I from *Anthopleura fuscoviridis* [39]; ATX Ia from *Anemonia sulcata* [40]. Cysteine residues are shaded, hydroxy-Pro residues are denoted by “O”, and identical residues with Hau IV are boxed. Asterisks under the sequence of ATX Ia represent the residues typical of only type 1 toxins.

Figure 3 shows the amino acid sequences of Hau I to Hau IV, along with those of homologous sea anemone peptide toxins found by the NCBI and UniProt BLST algorithm. As for Hau I (30 amino acid residues), no hits were returned in searching by the BLAST. In Figure 3 A , HC-18 (identical to Hau I, except for the difference at the fifth residue due to a post-translational modification) and HC-19 (analogue of HC-18) are aligned with Hau I. It is quite intriguing that both HC-18 and HC-19 have already been detected in the sea anemone *Heteractis crispa*, a close relative species of *H. aurora* used in this study, through a recent transcriptomic analysis by Guo *et al.* [25]. 

Hau II (27 amino acid residues) and Hau III (28 amino acid residues), which show 48% sequence identity with each other, were found to be members of the BBH family, such as SHTX I and II (both type 4 potassium channel toxins) from *S. haddoni* [16], Am I (possibly sodium channel toxin) from *A. maculata* [31], Sco 9a-1 (anti-inflammatory peptide) from *Stomphia coccinea* [33], Ms 9a-1 and MS9.1 (both modulators of TRPA1) from *Metridium senile* [34]*,* and Ugr 9a-1 (ASIC3 inhibitor) from *Urticina grebelnyi* [35], as aligned in Figure 3 B . The remaining one component, Hau IV (49 amino acid residues), was identified as a new member of the type 1 sodium channel toxin family, such as Cp I from *Condylactis passiflora* [36], Rc I from *Radianthus crispus* [37], Am III from *A. maculata* [31], ApA from *Anthopleura xanthogrammica* [38], AFT I from *Anthopleura fuscoviridis* [39], and ATX Ia from *Anemonia sulcata* [40], as aligned in Figure 3 C . However, two of the six residues typical of only type 1 sodium channel toxins [6] were replaced in Hau IV by other residues (Gly at position 2 and Trp at position 24). 

## Discussion

In this study, four peptide toxins (Hau I-IV) with crab toxicity were isolated from the sea anemone *H. aurora* using two chromatographic steps, gel filtration and reverse-phase HPLC, and their amino acid sequences were determined by a combination of protein sequencing and cDNA cloning. Importantly, not only lethal toxins (Hau I and IV) but also paralytic toxins (Hau II and III) were identified by careful observation of the symptoms induced in crabs. Structurally and functionally diverse crab-paralyzing peptide toxins, such as gigantoxin I (EGF-like toxin) from *S. gigantea* [20], Am II (peptide toxin of unknown function) from *A. maculata* [31], SHTX I and II (both type 4 potassium channel toxins) from *S. haddoni* [16], BcIV (possibly sodium channel toxin) from *Bunodosoma caissarum* [41], and PhcrTx2 (peptide toxin of unknown function) from *Phymanthus crucifer* [42], have so far been isolated from sea anemones and characterized to some extent. This study again confirms the usefulness of the crab assay for the discovery of novel peptide toxins in sea anemones. 

The significance of this study lies in the identification of Hau I, a novel class of peptide toxin, for which no similar peptide toxins were found in the BLAST search. Based on transcriptomic analysis, Guo *et al.* [25] reported that Hau I (HC-18) and its analogue (HC-19) are expressed in *H. crispa*, although they provided no information on their toxicity. In this study, we clarified that Hau I is one of toxic components contained in *H. aurora* by the conventional approach of chromatographic isolation and structural analysis. In recent years, transcriptomics, proteomics, and the integration of both omics have been effectively utilized for comprehensive analysis of toxic peptides and proteins contained in various species of sea anemones [5, 24-27]. However, omics approaches are rather vulnerable to unknown peptides and proteins, such as Hau I. In the case of unknown peptides or proteins, whether they are truly toxins must await further study on their expressed or chemically synthesized products. Therefore, in order to discover a number of novel toxic components hidden in sea anemones, conventional approaches such as this study, which involve steadily isolating toxic components and analyzing their structures, still remain important. 

Guo *et al.* [25] hypothesized that Hau I (HC-18) is a member of the BBH family and targets TRPA1, similar to MS9.1 [34]. However, this hypothesis seems unlikely. First, Hau I shows no sequence homology to BBH family peptide toxins including MS9.1, except for the cysteine ​​pattern CXXC-C-C. Second, Hau I exhibits strong lethal activity against crabs, whereas TRPA1 modulators such as MS9.1 are probably non-toxic to experimental animals as described later. We therefore assume that Hau I is a novel class of sea anemone peptide toxin independent of the BBH family. Although the strong crab-toxicity (LD_50_ of 14 μg/kg) suggests that Hau I acts on sodium channels, it should be noted that the symptoms induced in crabs by Hau I were considerably different from those by known sea anemone sodium channel toxins (including Hau IV in this study). The known sea anemone sodium channel toxins induce in crabs strong stiffening and thrust of walking legs following injection, whereas crabs challenged with Hau I did not exhibit such symptoms and died with the whole body relaxed. Since Hau I is a structurally novel peptide toxin that would be useful as a pharmacological reagent and also as a lead compound for developing therapeutic agents, future studies are needed to clarify whether or how Hau I affects sodium channels and/or other various types of channels. 

Hau II and III are homologous to each other and also to members of the BBH family, as aligned in Figure 3 B . The BBH family toxins are relatively short peptides and are characterized by having the same cysteine pattern CXXC-C-C but are diverse in terms of biological activity. Hau II and III show paralytic activity in crabs, although their potencies (ED_50_ of 2350 μg/kg for Hau II and 1400 μg/kg for Hau III) are significantly weaker than those (ED_50_ of 215-430 μg/kg) of the crab-paralyzing peptide toxins (SHTX I and II from *S. haddoni* [16], gigantoxin I from *S. gigantea* [20], and Am II from *A. maculata* [31]) that have previously been isolated from sea anemones. Unlike Hau II, Hau III, SHTX I, and SHTX II, Am I is lethal to crabs. Surprisingly, however, the difference in amino acid sequence between Hau II and Am I is recognized only at the C-terminus (Ala for Hau II and Ser for Am I). Future detailed studies should focus on the structure-activity relationship of Hau II, which will reveal why the replacement of only the C-terminal residue causes such a remarkable difference in crab toxicity between Hau II and Am I. It is also worth mentioning that, differing from Hau II and III, three TRPAI modulators (Sco 9a-1, MS 9a-1, and MS9.1) and one ASCI3 channel inhibitor (Ugr 9a-1) are probably non-toxic to experimental animals. In fact, Sco 9a-1 was shown to be non-toxic to mice [33], whereas Ugr 9a-1 was neither lethal nor paralytic to crustaceans (noble crayfish *Astacus astacus*) [35]. Taking all of the above into consideration, it can be concluded that Hau II and III are closest to SHTX I and II (potassium channel toxins) in terms of biological activity within the BBH family. Future studies are needed to evaluate whether both Hau II and III exhibit potassium channel toxicity, similar to SHTX I and II. 

Regarding Hau II and III, it is also worth mentioning that the precursor proteins contained multiple copies of the mature peptides (four and two copies for Hau II and III, respectively). All four copies in the Hau II precursor were commonly preceded by the same four residues (Glu-Pro-Glu-Pro) and followed by the same three residues (Arg-Lys-Arg). In the case of the Hau III precursor, one copy had four extended residues (Glu-Pro-Lys-Pro) at the N-terminus and three extended residues (Arg-Lys-Arg) at the C-terminus, however, the other copy was devoid of the three C-terminal residues in the precursor proteins. As in the case of Hau II and III, precursor proteins containing multiple copies of the mature peptide have been found in not all but many peptide toxins of the BBH family. For example, the precursor proteins of Am I [31] and Ugr 9a-1 [35] contain six and four copies of the mature peptide, respectively. Moreover, examples such as Ms 9a-1 [34], in which a single precursor protein contains different mature peptides (e.g. Ms 9a-1 and Ms 9a-2), are not uncommon. The presence of multiple copies of the same or different mature peptides within one precursor protein is undoubtedly a great advantage for sea anemones to rapidly produce large amounts of peptide toxins. 

Hau IV is a new member of the sea anemone type 1 sodium channel toxin family. Norton [6] noted that type 1 sodium channel peptide toxins are contained in members of the Actiniidae family, whereas type 2 toxins are present in members of the Stichodactylidae family. However, this study established the presence of a type 1 toxin in *H. aurora* that belongs to the family Stichodactylidae. Similarly, type 1 toxins have been found in three Stichodactylidae species: *R. crispus* [37], *S. gigantea* [20] and *A. maculata* [31]. Interestingly, both type 1 and 2 toxins have been detected in *S. gigantea*. Taken together, it is reasonable to consider that members of Stichodactylidae contain either type 1 or 2 toxins or both.

In this study, Hau I-IV in *H. aurora* were isolated using the whole organism and hence their tissue distribution is unknown. In this regard, Guo *et al.* [25] reported an interesting finding that Hau I (HC-18) is expressed in the body column and mesenterial filaments of *H. crispa*, but not in the tentacles. This tissue distribution of Hau I is naturally applicable to *H. aurora*. Therefore, the function of Hau I in both *H. crispa* and *H. aurora* can be inferred as follows: (1) Hau I is not involved in the capture of prey animals, such as crustaceans and small fish, by the tentacles, (2) Hau I in the body column serves as the defenses against predators, (3) Hau I in the internal mesenterial filaments is used to paralyze and kill prey animals that has been captured and taken into the body, and (4) Hau I in the mesenterial filaments released outside the body is used in external defense and competition. To understand the ecological function of the peptide toxins in *H. aurora* in more detail, future studies are needed to clarify their tissue distribution as well as their pharmacological activities.

## Conclusions

This study revealed that a novel class of toxin (Hau I), two BBH family toxins (Hau II and III), and a type 1 sodium channel toxin (Hau IV) are present in *H. aurora*. Hau I-III are expected to be novel pharmacological agents; therefore, future studies on their modes of action are required. Furthermore, the isolation and characterization of peptide toxins from various species of sea anemones should continue to discover structurally and functionally unique peptide toxins. 

## Abbreviations

ED_50_: effective dose, 50 %; HPLC: high performance liquid chromatography; LD_50_: lethal dose, 50 %; MALDI/TOFMS: matrix assisted laser desorption ionization/time-of-flight mass spectrometry; RACE: rapid amplification of cDNA ends; RT-PCR: reverse transcription PCR; TFA: trifluoroacetic acid. 

## Data Availability

All data generated or analyzed during this study are included in this published article.
